# Closed-loop control of a fragile network: application to seizure-like dynamics of an epilepsy model

**DOI:** 10.3389/fnins.2015.00058

**Published:** 2015-03-03

**Authors:** Daniel Ehrens, Duluxan Sritharan, Sridevi V. Sarma

**Affiliations:** Neuromedical Control Systems Lab, Biomedical Engineering, Institute for Computational Medicine, Johns Hopkins UniversityBaltimore, MD, USA

**Keywords:** closed-loop stimulation, neuronal network model, network fragility, epilepsy model, hidden Markov models

## Abstract

It has recently been proposed that the epileptic cortex is *fragile* in the sense that seizures manifest through small perturbations in the synaptic connections that render the entire cortical network unstable. Closed-loop therapy could therefore entail detecting when the network goes unstable, and then stimulating with an exogenous current to stabilize the network. In this study, a non-linear stochastic model of a neuronal network was used to simulate both seizure and non-seizure activity. In particular, synaptic weights between neurons were chosen such that the network's fixed point is *stable* during non-seizure periods, and a subset of these connections (the most fragile) were perturbed to make the same fixed point *unstable* to model seizure events; and, the model randomly transitions between these two modes. The goal of this study was to measure spike train observations from this epileptic network and then apply a feedback controller that (i) detects when the network goes unstable, and then (ii) applies a state-feedback gain control input to the network to stabilize it. The stability detector is based on a 2-state (stable, unstable) hidden Markov model (HMM) of the network, and detects the transition from the stable mode to the unstable mode from using the firing rate of the most fragile node in the network (which is the output of the HMM). When the unstable mode is detected, a state-feedback gain is applied to generate a control input to the fragile node bringing the network back to the stable mode. Finally, when the network is detected as stable again, the feedback control input is switched off. High performance was achieved for the stability detector, and feedback control suppressed seizures within 2 s after onset.

## 1. Introduction

Epilepsy is a neurological condition that affects approximately 70 million worldwide. Incidence of this condition is twice as frequent in low and middle income countries than in those with high income (Ngugi et al., 2011). Approximately 20–30% of the population with epilepsy suffers from intractable epilepsy and must consider invasive alternatives such as resective surgery, vagal nerve stimulation, and deep brain stimulation therapy (Schuele and Lüders, 2008). Recently, implanted responsive or closed-loop neurostimulators (RNS, NeuroPace, Mountain View, California) were FDA approved (Fisher and Velasco, 2014). Such closed-loop stimulation systems for epilepsy rely on early and accurate detection of the seizure onset in order to be able to disrupt the seizure before clinical manifestations occur (Santaniello et al., 2011); and, reliable detection of seizure onsets require understanding the electrophysiological dynamics in the cortical epileptic network.

Seizures are characterized by abnormal electrical activity generated by large neuronal populations (Uhlhaas and Singer, 2006), electrical brain stimulation for the treatment of epilepsy focuses on suppressing the abnormal activity (Sohal and Sun, 2011; Fridley et al., 2012). Single unit recordings show that during seizures there exists heterogeneity in neuronal firing patterns, where firing rate may either increase, decrease or vanish altogether (Truccolo et al., 2011). This heterogeneity at the neuronal level, however, evolves into a highly synchronized behavior at the network level. The complex network dynamics seen in seizures have led to several hypotheses for the etiology of epilepsy, which still remains unclear. Studies have implicated axo-axonic gap junctions (Traub et al., 2001), loss of inhibitory chandelier cells in cortex (DeFelipe, 1999), atypical axonal sprouting from layer V pyramidal cells (Jin et al., 2006), and neurotransmitter imbalance (Bradford, 1995) among others. A shared thread among proposed etiologies is that coupling within the neuronal network is altered, resulting in epileptiform spiking. This suggests that epilepsy could be a network-driven phenomenon in which the structural connectivity between neurons is altered rendering unstable pathological functional activity.

Computational modeling is a powerful tool for understanding the underlying mechanisms involved in seizure genesis and its dynamics, and has proven to be very useful in aiding with the design of more efficient seizure onset detectors and brain stimulation protocols (Stefanescu et al., 2012). We recently constructed a neuronal network model of the epileptic cortex that qualitatively captures the heterogeneity in neurons observed in patients during seizure events (Sritharan and Sarma, 2014). In Sritharan and Sarma (2014), it is posited that the epileptic cortex is on the brink of instability and that small perturbations in the synaptic connections render the network unstable temporarily. That is, the epileptic cortex is constantly transitioning (albeit maybe not so frequently) between a stable state (non-seizure) and an unstable state (seizure). Therefore, in Sritharan and Sarma (2014), the neuronal network model used operates in two modes: stable and unstable, distinguished only by the synaptic weights to a single fragile node. Nodal *fragility* is defined as the minimum energy perturbation in functional connectivity that renders the entire network unstable.

The goal of this study is to design a feedback control system that first detects when the network has gone unstable from neuronal spike train measurements, and then applies a gain to the measured firing rates to generate a control input to the network. The two-state (seizure mode, non-seizure mode) model described in Sritharan and Sarma (2014) and Ehrens et al. (2014) is used to design the feedback controller which applies an exogenous perturbation (e.g. stimulation) to the most fragile node in the network in order to return the network to its stable mode. The controller is then shut off once the network is detected as having been stabilized.

More specifically, the stability detector in the controller uses a two-state (stable, unstable) Hidden Markov Model (HMM) to represent the non-linear neuronal network dynamics. The fragile neuronal network model from Sritharan and Sarma (2014) is used to simulate firing rate activity of the most fragile node (the HMM observations) in each mode, and this activity is modeled as a Gaussian random variable whose mean and variance are determined through maximum likelihood estimation. Therefore, the output or emission probabilities of the HMM are each Gaussian, and the transition probability from the stable mode to the unstable mode is fixed, rendering a geometric distribution representing the time until seizure onset.

In order to detect when the network has transitioned from a stable to an unstable mode, an algorithm we recently developed based on the HMM (Ehrens et al., 2014) was implemented. Specifically, the derivative of the cumulative likelihood ratio is first computed from firing rate measurements generated by the non-linear network model and the Gaussian distributions of the HMM, and then when this derivative exceeds a certain threshold, the transition to unstable mode is detected. Different thresholds and different scenarios were considered for computing the firing rate of the fragile node, until a high performing (i.e., minimal number of false positives and small delays between the seizure onset and its detection) detector was found. See (Ehrens et al., 2014) for details.

A feedback controller is activated when the transition to the unstable mode is detected. The controller consists of a gain that acts on the measured firing rates of all nodes in the network, and generates a scalar control input to the most fragile node in the network. While the control is switch “on,” the stability detector continues to monitor the firing rate of the fragile node. When the average firing rate of the fragile node returns to its baseline rate in the stable mode (i.e. within two standard deviations of this baseline) and stays there for at least 500 *ms*, the detector turns the control input “off,” and continues to monitor the network activity to detect the transition to the unstable mode again.

Two types of feedback controllers were derived and compared in their performance. One of the feedback controllers is based on the nonlinear neuronal model and the other is based on a linearization of the model. Simulation of the epileptic neuronal network restated the high performance of our instability detection algorithm (Ehrens et al., 2014). Similarly, efficiency results showed that both types of feedback controllers were able to return the neuronal network to its stable mode in less than 2 s.

The proposed approach assumes that the state of each neuron in the network is available at all times, as well as the synaptic weights of the network. Also *a priori* knowledge of which is the most fragile node in the network is assumed in order to deliver state feedback to this node. These assumptions and the difficulty of targeting single neurons to deliver electrical stimulations are limitations for the implementation of the proposed protocol in real-physiological conditions. Nevertheless, the feedback controller architecture proposed here may be extended to measuring and stimulating populations of neurons.

## 2. Materials and methods

**Figure 1:**
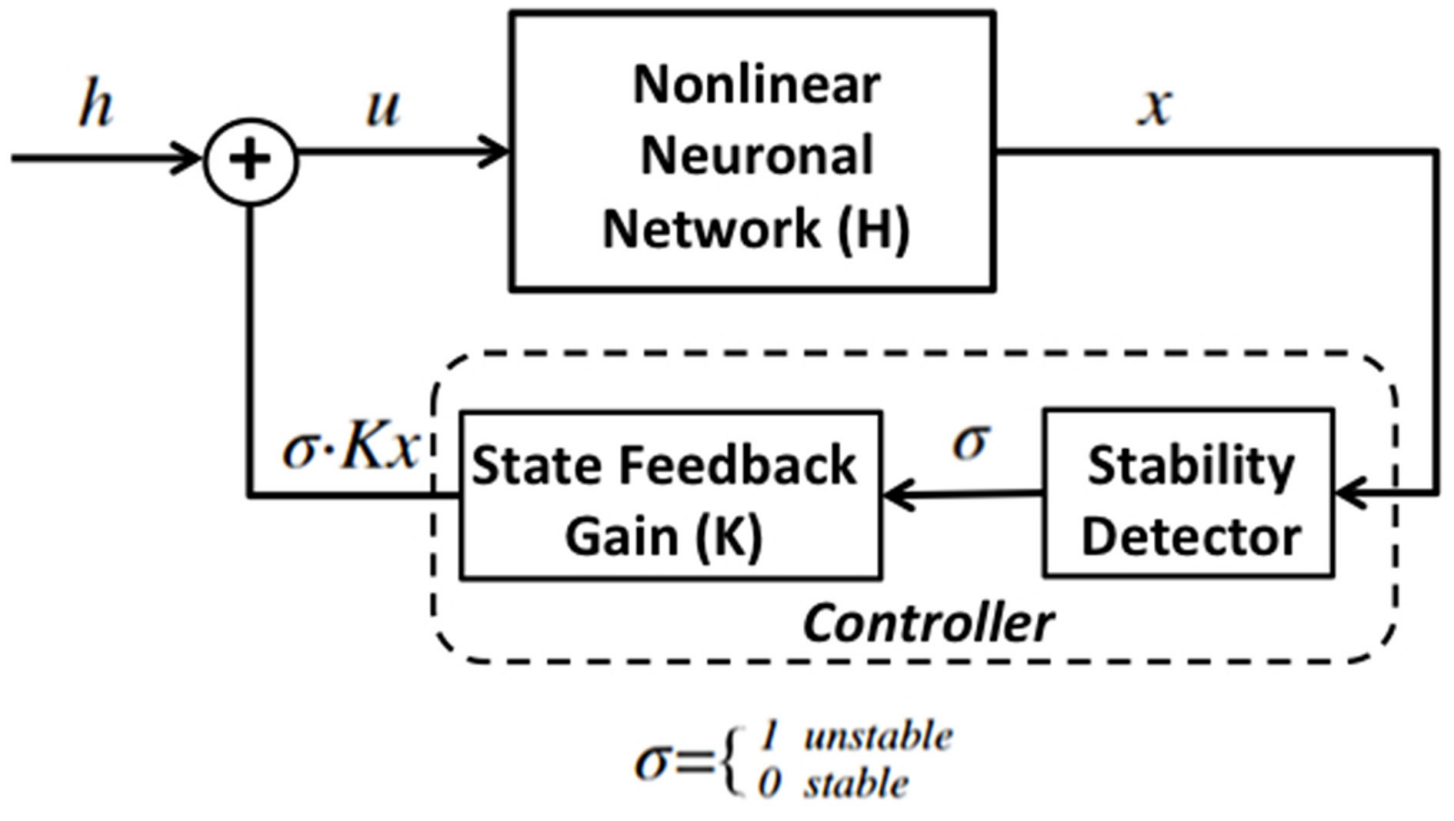
**Diagram for state feedback control of the proposed system**. H is a nonlinear neuronal network that switches between unstable and stable modes, has external input *u* and output *x* which is the state vector of the network and is used to compute the firing rate of the network. The controller is composed of a stability detector that governs the activation of a state-feedback gain.

This section first describes the non-linear model of epileptic neuronal network (Sritharan and Sarma, 2014), and then describes the feedback controller architecture which consists of the stability detector and a state-feedback gain. Figure 1 shows a diagram of the control system. The controller is regulated by the stability detector. When σ = 1 the detector believes that the network is unstable and turns the controller “on,” and when σ = 0 the network is assumed stable and the controller is shut “off.”

### 2.1. The neuronal network model

**Figure 2:**
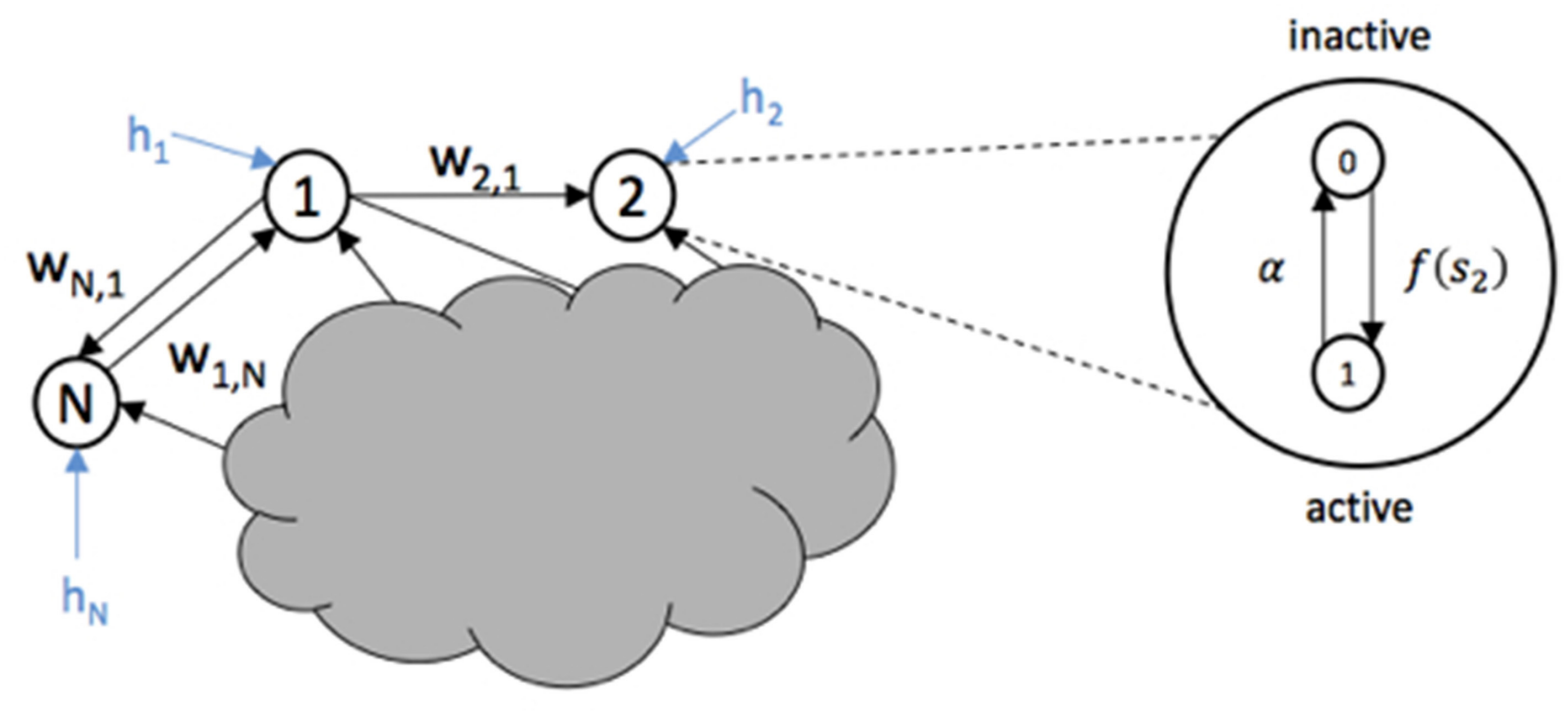
**Three nodes from a network are shown with weight edges represented by *w_ij_* and external input *h_i_***. The internal activation-inactivation structure of node 2 is shown as a Markov process where the transition to an active state (1) is given by *f*(*s*_2_) and the transition to inactive state (0) is given by α.

The system to be controlled is the epileptic network and is modeled by a non-linear stochastic network model as the one proposed in Benayoun et al. (2010). The model consists of a set of *N* interconnected nodes, with edge weights that represents synaptic strengths between pairs of nodes. See Figure 2. Each node can represent a single neuron or a population of neurons. However, in this study, it is assumed that each node is a single neuron.

The internal activity of each node depends on its synaptic inputs and its current firing state. Let node *i* be a neuron that at some time *t* is either active (*x_i_*(*t*) = 1) or quiescent (*x_i_*(*t*) = 0). Then, the transition between active and inactive states evolves as a Markov process with rate constants for a small time interval Δ*t*. That is,

(1) $$Pr\;\left\{\left(x_{i}\left(t+\Delta t\right)=0;x_{i}\left(t\right)=1\right)\right\}=\alpha \Delta t$$

(2) $$Pr\;\left\{\left(x_{i}\left(t+\Delta t\right)=1;x_{i}\left(t\right)=0\right)\right\}=f\left(s_{i}\left(t\right)\right)\Delta t$$

A node being active represents an action potential, including its refractory period. As shown above, neuron *i*'s propensity to transition from inactive to active depends on its total synaptic input, represented by *s_i_*(*t*) defined below. The propensity to transition from active to inactive, however, is constant and thus a neuron on average is active for a period of α^−1^. *f* is a non-linear response function that represents the firing rate of a node when quiescent. For simulation purposes, a clamped hyperbolic tangent was used as in Sritharan and Sarma (2014). From (1) and (2), the probability of a neuron *i* being active at any time *t* evolves according to the following non-linear rate equation:

(3) $${\dot{r}}_{i}(t)=-\alpha r_{i}\left(t\right)+f\left(s_{i}\left(t\right)\right)\left[1-r_{i}\left(t\right)\right]$$

The network of *N* nodes is parameterized by the structural connectivity matrix, **W** = [*w_ij_*]. Each element in **W** describes the effect of node *j* on node *i*. Positive values represent excitation, negative values represent inhibition and a zero value means there is no connection between nodes *j* and *i*. The total synaptic input to node *i*, *s_i_*, depends on the state of the nodes that are connected to node *i*, the weight in its connections, and an external input, *u_i_*. The external input *u_i_* is given by the sum of *h_i_* (fixed value that represents background activity) and the feedback control input (discussed later). If node *j* is active, the synaptic input on node *i* is either increased or decreased.

The synaptic input is given by:

(4) $$s_{i}(t)=\sum_{j=1}^{N}w_{ij}x_{j}(t)+u_{i}$$

It has been shown that a stable fixed point, *r* ∈ ℝ^*N*^, exists in this model (Sritharan and Sarma, 2014). Then *r* is a steady state probability that satisfies *g*(*r*; **W**) = 0 and represents the baseline behavior of the network. It is shown in Sritharan and Sarma (2014) that *r* can be computed through a gradient descent algorithm that iterates over candidate solutions to minimize a cost function.

Note that (3) estimates the functional activity of the network given some network structure, **W**. Then, the result of linearizing Equation (3) around the fixed point, *r*, is the functional connectivity matrix, **A**. Therefore, **A** has eigenvalues λ_1…*N*_ ∈ ℂ where ℝ^*N*^ {λ_1_} ≥ … ≥ ℝ^*N*^ {λ_*N*_}. The functional connectivity matrix captures how the probability of any node being active affects the probability of node *i* being active. Since *r* is a stable fixed point, ℝ λ{_*i*_(**A**)} < 0∀*i*.

In Sritharan and Sarma (2014), the minimum energy functional perturbation that destabilizes the network is determined and then the structural changes that would produce this functional perturbation are derived. The minimum energy perturbation is modeled by adding a perturbation matrix Δ to **A**, where Δ has only one non-zero row. This represents a change in the inbound effect of the network on that node. The minimum perturbation takes ℝ^*N*^ {λ_1_(**A** + Δ)} = 0 where the system would be marginally stable. A larger perturbation can make ℝ^*N*^ {λ_1_(**A** + Δ)} > 0 which is still unstable. In Sritharan and Sarma (2014), it is shown how the minimum energy Δ can be computed using least squares.

To build the model of a neuronal epileptic network, the connectivity matrices derived in Sritharan and Sarma (2014) were used. Three connectivity matrices were used; one functionally stable structural matrix (**W**_*s*_) and two functionally unstable structural matrices (**W**_*u*0_, **W**_*u*200_), where ℝ^*N*^ {λ_1_(**W**_*u*0_)} = 0 ms^−1^ and ℝ^*N*^ {λ_1_(**W**_*u*200_)} = 200 ms^−1^. An non-seizure stable mode is simulated by using **W**_*s*_ in (3), and an unstable seizure mode is simulated by using either **W**_*u*0_ or **W**_*u*200_.

The model simulation follows the Gillespie stochastic algorithm (Gillespie, 1977) with the following steps for a network with *N* nodes.:

1. Pick initial conditions for the nodal states, *x_i_*(0).
2. Evaluate synaptic inputs using Equation (4).
3. Calculate transition rates using:
  $$tr_{i}(t)=\alpha x_{i}\left(t\right)+f\left(s_{i}\left(t\right)\right)\left[1-x_{i}\left(t\right)\right]$$
4. Determine the network transition rate:
  $$tr_{net}=\sum_{i=1}^{N}tr_{i}$$
5. Switch the state of a single node *i* with probability *tr_i_*/*tr_net_* and update *x*(*t*).
6. Draw timestep Δ*t* from an exponential distribution, where *n* ~ *N*(0,1) and Δ*t* = −*log*(*n*)/*tr_net_*.
7. Increment time by Δ*t* and repeat from step 2.

**Figure 3:**
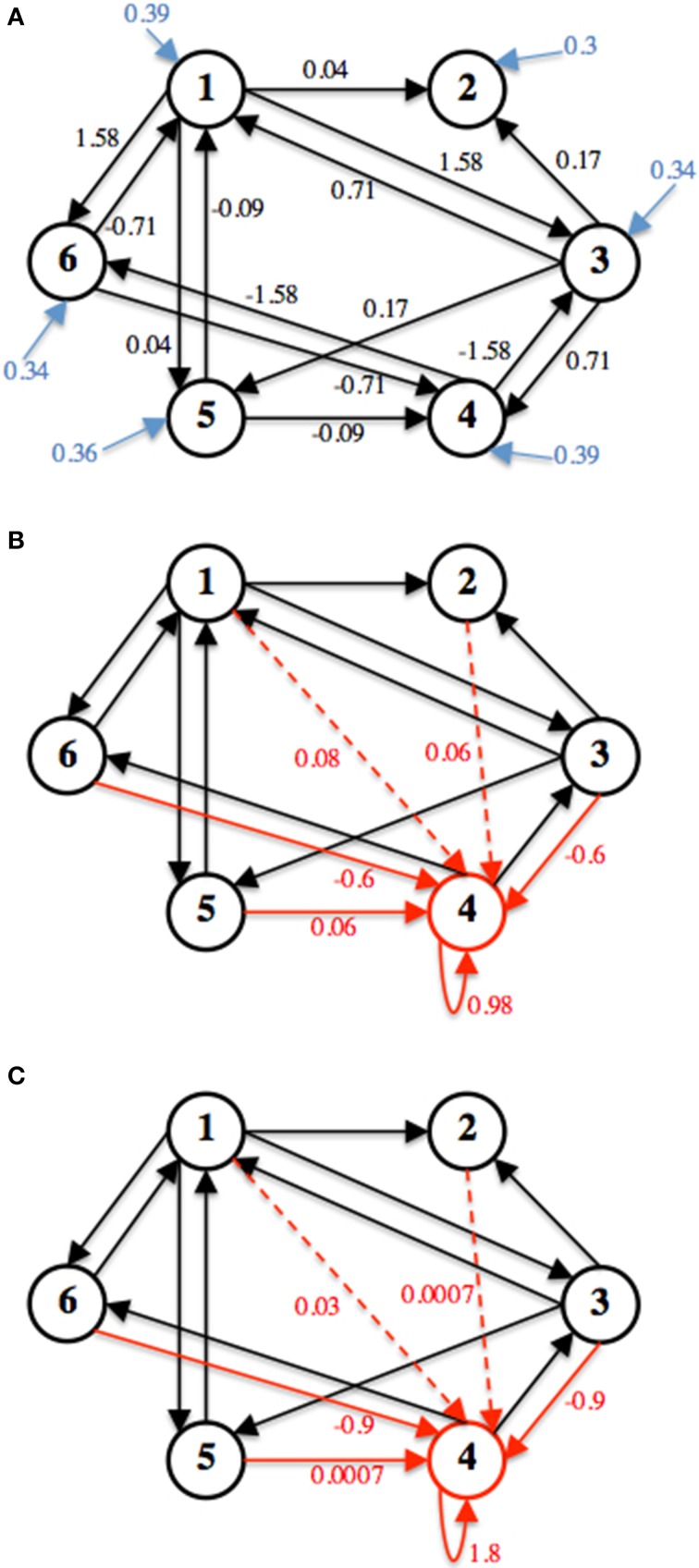
**The three connectivity matrices used for simulation are shown**. **(A)** Shows **W**_*s*_, black arrows represent weight edges (*w_ij_*), blue arrow represent external input (*h_i_*). **(B)** Shows the computed Δ to acquire **W**_*u*0_ and **(C)** Shows the computed Δ to derive **W**_*u*200_. Δ is represented by the red arrows in **(B)** and **(C)** respectively.

The network simulated here has 6 nodes, within each node is a single neuron, and 14 connections. Figure 3 shows the weight edges and external input for the functionally stable connectivity matrix (**W**_*s*_). Also in Figure 3, two different row perturbations are shown, from these perturbations **W**_*u*0_ and **W**_*u*200_ are derived and used to simulate the unstable seizure mode.

The decay rate, α, is set to 100 Hz, which caps the neuronal firing rates at that value. Both **W**_*u*0_ and **W**_*u*200_ have a row perturbation at a DC frequency on node 4, an inhibitory neuron. Therefore, when the unstable mode of the neuronal network is simulated, the firing rate of this neuron decreases, thus producing disinhibition in the network which increases firing activity in the network. All simulations were done using MATLAB.

### 2.2. HMM representation of the neuronal network

**Figure 4:**
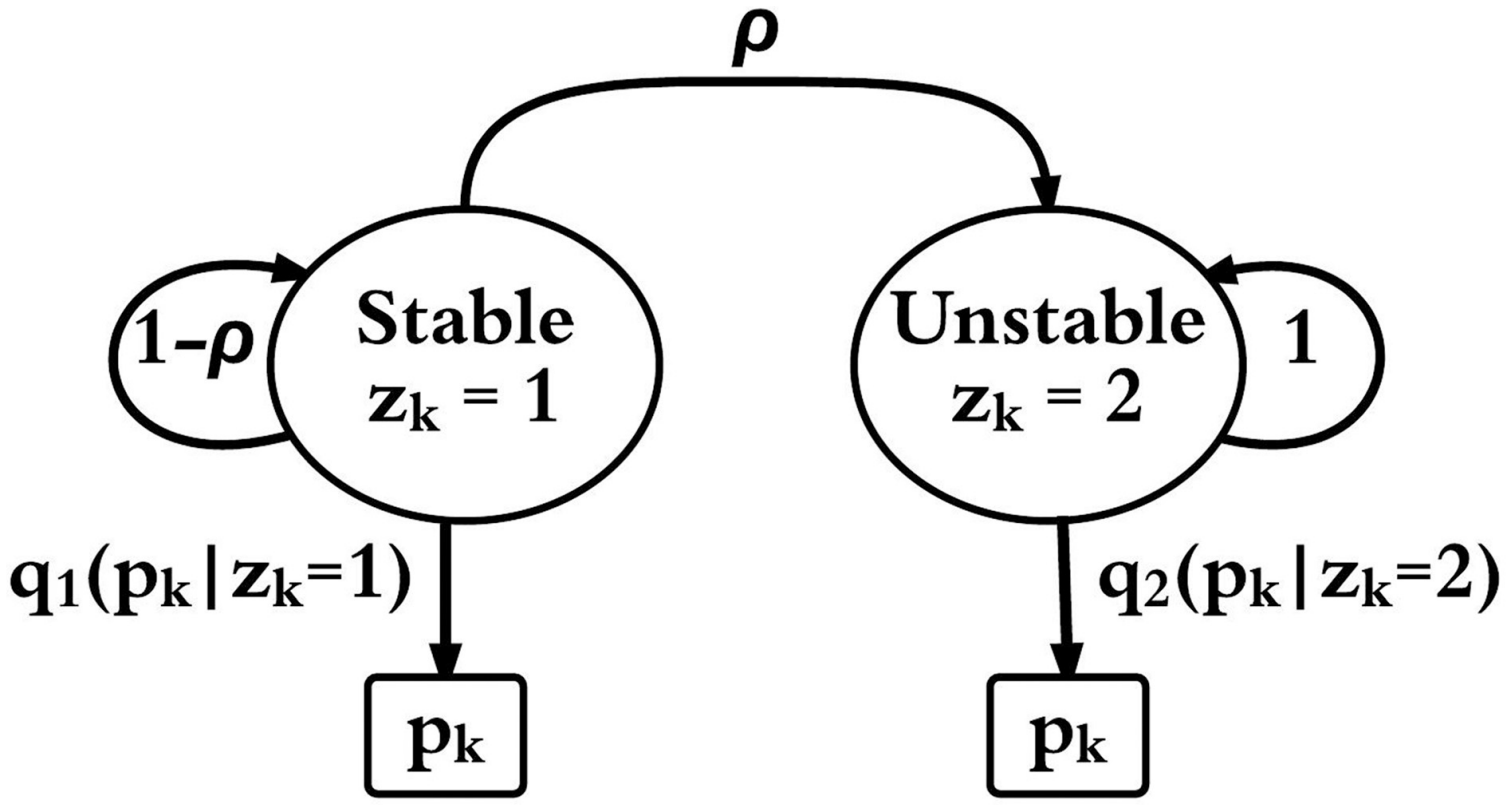
**HMM schematic with two modes (*z_k_* = 1) and (*z_k_* = 2) and observable output** *p_k_*. *q_z_*(*p_k_*) is the probability function of *p_k_* in mode *z* ∈ {1, 2} and ρ is the probability of transition from mode 1 to mode 2.

A two-mode HMM is constructed to represent an epileptic neuronal network. Figure 4 shows a schematic of the HMM. The output observation is *p_k_*, which is the firing rate of the most fragile node in the original non-linear network described above, and it is obtained at discrete time steps *k* = 0, 1, 2, … computed from the system's state vector; *x_k_*. Note that the actual time step is given by Δ*t* as described above. The network is in one of two modes at each time step; *k* a stable non-seizure mode (*z_k_* = 1) or an unstable seizure mode (*z_k_* = 2). The initial mode is always assumed as stable, that is, *z*_0_ = 1 and it transitions to the unstable mode with a fixed probability ρ = 0.0002. The HMM does not include a transition back to the stable mode. Once the feedback gain is activated and a stable firing rate is detected then **W**_*s*_ is used to simulate the stable mode again.

The output density functions *q*_1_ and *q*_2_ are assumed to be Gaussian, and the parameters for these functions were computed using a maximum likelihood estimation from a 100 s simulation of each mode from the original non-linear model.

### 2.3. Feedback controller

The proposed closed-loop control architecture is composed of the stability detector and the state-feedback gain matrix. When the system is in the stable mode (*z_k_* = 1), σ = 0 and the state-feedback gain is turned off. The detector takes in firing rate measurements from the fragile node in the non-linear network to detect a transition to the unstable mode (*z_k_* = 2). Similarly, when the system is unstable (*z_k_* = 2), σ = 1 and the state-feedback gain is activated in order to return the firing rate to its baseline. The detector then takes firing rate measurements from the fragile node in the non-linear network to detect the transition back to a stable mode (*z_k_* = 1).

#### 2.3.1. Stability detector

When σ = 0, *p_k_* is computed every time step, Δ*t*, using the spike train of the fragile node. Specifically, it retrospectively counts the number of spikes in an immediate fixed time-length window, *m*, that shifts with every Δ*t*. Then, the number of spikes is divided by the maximum number of spikes for that window size, to obtain the firing rate. The maximum number of spikes for each window length was obtained from a 100 s simulation of the network in the stable mode. Finally, the firing rate is averaged for *n* of the most recent windows. The detector and the chosen set of parameters (*m*, *n*) are discussed in more detail in our recently developed stability detector (Ehrens et al., 2014). In this study, a high performance detector with minimum delay was used where the window size is *m* = 250 *ms*, and the firing rate is averaged across the past *n* = 25 windows.

The architecture of the detector implemented has two components: a cumulative likelihood generator and a threshold classifier. From measurements of *p_k_* and the HMM emission distributions, the likelihood ratio is computed as follows:

$$LR_{k}\equiv q_{2}\left(p_{k}\right)/q_{1}\left(p_{k}\right)$$

When *LR_k_* > 1, *p_k_* is more likely to belong to *q*_2_ and hence the network is more likely to be unstable. Once *LR_k_* is computed, the detector computes the LR cumulative as:

$$gr_{k}=\sum_{l=1}^{k}LR_{l}$$

The cumulative sum captures if *p_k_* has been more likely to belong to *q*_2_ (*LR_k_* >> 1). If this is the case, then *gr_k_* will significantly increase.

**Figure 5:**
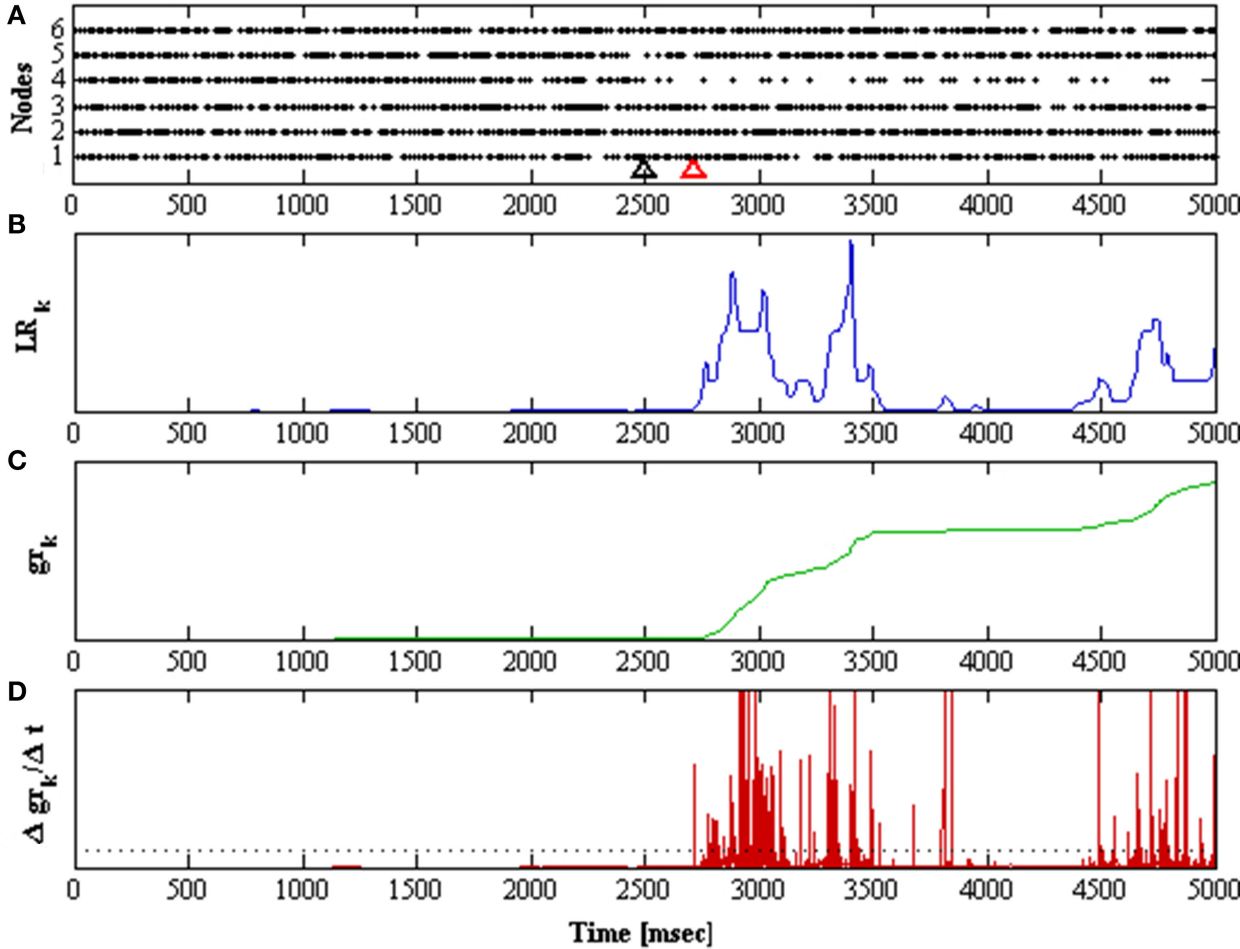
**Shows a 5 second simulation of the network**. The network is initially in stable mode and at *t* = 2.5 s it is perturbed, taking λ_1_ to 200 ms^−1^. **(A)** Raster plots for all nodes in the network. The black arrow marks when the perturbation was applied, the red arrow marks when it was detected. **(B)** Likelihood ratio of the observation distributions over time. **(C)** The cumulative function *gr* of the likelihood ratio over time. **(D)** The derivative of the cumulative function over time.

In order to determine if *gr_k_* shows a rapid increase, the detector takes its derivative (Δ*gr_k_*/Δ*t*), and detects if there is a sudden change indicating that *p_k_* is generated from *q*_2_. Detection of a transition to an unstable mode occurs when the derivative of the cumulative likelihood ratio exceeds a threshold value. The threshold was obtained from the mean average value of the (Δ*gr_k_*/Δ*t*) over a 60 s simulation of both functionally unstable networks; **W**_*u*0_ and **W**_*u*200_. Figure 5 shows a sample detection of a transition unstable mode and illustrates the behavior of the detector components.

If a transition to the unstable mode is detected and σ = 1, the detector then looks for a transition back to the stable mode produced by the feedback gain control. Stable mode detection occurs when the firing rate of the most fragile node in the network returns to its stable firing rate mean value and stays there for 500 *ms* within a range of two standard deviations around the stable firing rate mean value. When stability is detected, then σ = 0, and the state-feedback control is deactivated and **W**_*s*_ is used to simulate the stable mode again, and the transition back to *z_k_* = 1 in the HMM is imposed.

#### 2.3.2. State feedback gain (*K*)

The binary state of all the nodes in the network, *x*, is being observed, and thus the probability of each state being active, *r*, can be estimated. Recall that by plugging Equation (4) into (3) the evolution of each element in vector *r* is described as follows:

(5) $${\dot{r}}_{i}(t)=-\alpha r_{i}\left(t\right)+f(\sum_{j=1}^{N}w_{ij}x_{j}\left(t\right)+u_{i})[1-r_{i}\left(t\right)].$$

To construct the output probability distributions of the HMM, we linearize the system of Equation (5) to obtain the following system of equations

(6) $$\dot{r}(t)=Ar(t)+Bu(t),$$

where *r*(*t*) ∈ ℝ^6×1^, the input vector *u*(*t*) ∈ ℝ^6×1^, and the input matrix *B* ∈ ℝ^6×6^. *B* is obtained by linearizing Equation (5) with respect to *u_i_* around a stable fixed point that satisfies *g*(*ṙ*, *u*(*t*); **W**) = 0. Since the input *u*_*i*_ represents external stimuli to each node *i*, the input matrix *B* only has non-zero values along its diagonal.

The linearized network model is a continuous-time linear time-invariant system. The state-space description of the open-loop system when the controller is not activated and when the system is stable is given by (6). When the system is in the unstable seizure mode, the linearized network model from Equation (6) changes to:

(7) $$\dot{r}(t)=(A+\Delta )r(t)+Bu(t).$$

The closed-loop system, i.e., when the controller is activated, generates a control input *u* = σ · *Kx* + *h*. As mentioned *h* has a fixed value and represents background activity. Then by substituting *u* in Equation (7), and taking into account σ = 1, results in the following closed-loop state-space model of the network:

(8) $$\dot{r}(t)=(A+\Delta +BK)r(t)+Bh(t),$$

where *K* is the gain matrix to be designed. The goal of the feedback controller is to return the network back to its original stable mode. In order to accomplish this, *K* must satisfy

(9) A=A+Δ+BK.

Since Δ is known, then from (9) the state feedback gain is given by *K* = −*B*^−1^ · Δ. Since *B* is a diagonal matrix and the perturbation matrix Δ ∈ ℝ^6×6^ only has non-zero values in the fourth row, as explained above and in Sritharan and Sarma (2014), then *K* ∈ ℝ^6×6^, where the only non-zero values in the matrix are in its fourth row. With this, the system is taken back to its original stable state before the row perturbation (Δ) was applied, returning the firing rate of the most fragile node to its stable mode baseline.

The resulting *K* attempts to return the perturbed system back to its stable mode using the linearized model. One can also design the control input directly from the non-linear model described by Equation (5). During the unstable mode, i.e., [*w_ij_*] = [**W**_*s*_ + Δ]_*ij*_, an input with the same magnitude and inverse sign as the perturbation matrix used would take the system back to its original stable mode, making *K* = − Δ, resulting in *u_i_* = −Δ*ij* · *x_j_*(*t*) + *h_i_*. This cancels out the perturbation made in the functional connectivity matrix and takes the system back to its stable mode.

Performance of the feedback control based on the linearized model was compared to that of the feedback control based on the nonlinear model. Efficiency was evaluated based on the time it took to detect the stable mode, and the time it took to return the fragile node's firing rate to its stable baseline average for the first time. Both time periods were computed in relation to the detection of the unstable mode.

## 3. Results

In this study, it is assumed that access to the spike trains of the network are available. This is required in order to compute the parameters of the HMM's emission densities, *q*_1_ and *q*_2_, and the optimal parameters for the detector.

For the detector proposed in this study, two types of delays were encountered; a detection delay, defined as the time between the actual transition to the unstable mode and its detection; and an initial delay due to window averaging when computing the firing rate of node 4 (i.e., the fragile node in our example). This is the amount of time that the detector needs at the beginning of the simulation before being able to detect a transition. Both delays depend on the parameters used for the detector, their effects on the detector's performance were analyzed in Ehrens et al. (2014).

After the transition to the unstable mode is detected (*z_k_* = 2, σ = 1), the state feedback controller is activated. This takes the unstable network back to its stable fixed point. While the state feedback controller is activated, firing rate of the most fragile node is measured to detect when it returns to a stable firing rate and stays there for 500 *ms*. If the firing rate of the fragile node stays at its stable average for at least 500 *ms*, then it is assumed that the neuronal network has returned to its stable mode around a fixed point (*z_k_* = 1, σ = 0) and **W**_*s*_ is used again to simulate spiking activity.

**Figure 6:**
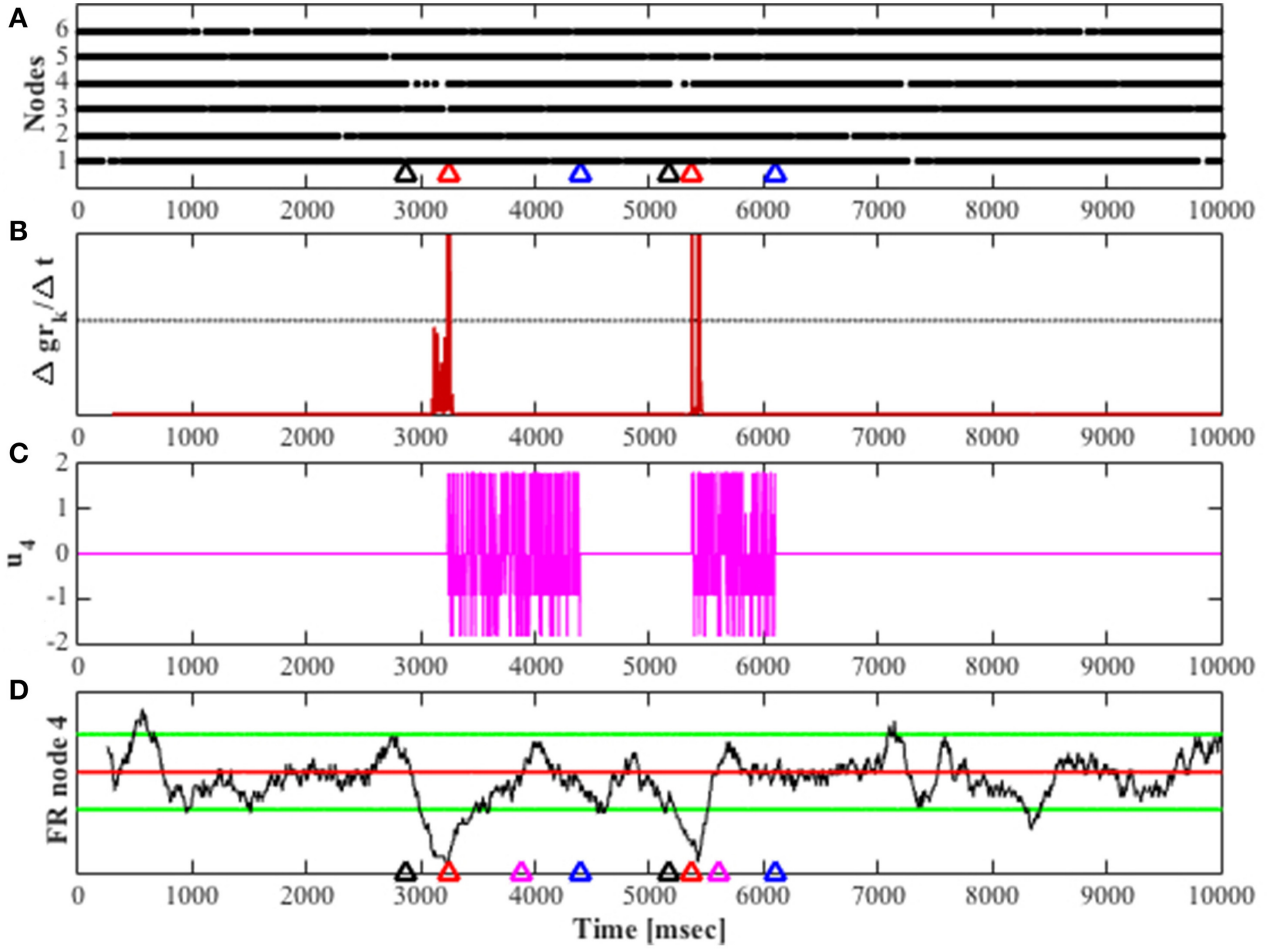
**Shows a simulation for 10 s, **W**_*u*200_ was used for unstable simulation**. **(A)** Raster plot for all nodes in the network. Color-coded arrows show mode transition and detection; black represents the transition to unstable mode, red marks the detection of the unstable mode and blue marks the detection of stability in the network. **(B)** The derivative of the cumulative function (Δ*gr_k_*/Δ*t*) over time, the black horizontal line marks the threshold for detection. **(C)** The feedback gain over time; input to the system's most fragile node, given by *u*. **(D)** The black trace shows the firing rate of node 4 over time. The red horizontal line shows the mean average value of stable mode firing rate, the green horizontal lines encompass the stable firing rate range with a value of twice the standard deviation above and below the mean average for stable mode firing rate. The magenta arrow marks when the fragile node returns to its baseline firing rate for the first time, other arrows are color-coded as in **(A)**.

The successful detection and control of the fragile neuronal network is shown in Figure 6, where the network transitions to an unstable mode twice, and each transition after being detected results in the activation of the feedback gain control. The firing rate of the most fragile node is also shown in this figure, and it is clear that it decreases when it becomes unstable and then increases when the feedback gain control is activated.

The feedback control performance was evaluated based on the time it took to detect the stable mode (stable mode delay), and the time it took to return the fragile node's firing rate to its stable baseline average for the first time (**1***st* stable FR), marked in Figure 6 by the magenta and red arrows, respectively.

Feedback control performance was analyzed by simulating the epileptic neuronal network model until 100 detections were done for each unstable matrix (**W**_*u*0_ and **W**_*u*200_), to test the robustness of the controller. All 400 detections to the unstable mode were true positives, with a mean average value of 406.9 ± 204.91 *ms* for the detection delays. The results for the controller performance are shown in Table 1, where we show the detection delays and the two time periods used for evaluating the efficiency of the feedback controllers.

**Table 1:** **Feedback Controller Performance**.

**Controller type**	**Stable mode delay[*s*]**	***1st* Stable FR[*ms*]**
Linear model	1.81±1.268	197±68.48
Non-linear model	1.05±0.49	307.2±166.94

From Table 1, it is clear that when feedback control is applied to the non-linear model the controller has a significantly smaller stable mode delay which is to be expected since the linearization of the model is an approximation that is not entirely accurate. However, the **1***st* stable FR period is smaller when applying feedback control to the linear model. The simulation results showed that both feedback controllers take back the neuronal network to its stable mode in less than 2 s.

## 4. Discussion

This study presents the design of a feedback control system applied to a recently developed model of a *fragile* epileptic network (Sritharan and Sarma, 2014). The behavior of the implemented non-linear stochastic neuronal network was manipulated by modifying the synaptic weights between neurons. The model assumes that epilepsy is a neuronal network condition, with random transitions between a stable (non-seizure) mode, and an unstable (seizure) mode, arising due to a perturbation to the most fragile node in the epileptogenic network.

The proposed control architecture measures the spike train observations from the epileptic network to detect when the neuronal network has transitioned from a stable to an unstable mode, marking the seizure onset, to then apply feedback control to the network to take it back to its original stable mode. The detector implemented is based on a recently developed 2-state HMM (Ehrens et al., 2014) that minimizes detection delay and maximizes the detection performance. In this study, the high performance of this detector was confirmed, since all detections (400) to the unstable mode were true positives, with a mean average value of 406.9 ± 204.91 *ms* for the detection delay. A limitation to the detection algorithm used in this study is that it requires obtaining the firing rates in each state *a priori*.

Two feedback control architectures were proposed, both able to return the neuronal network back to a stable mode after the neuronal network was perturbed. Performance of the feedback control was evaluated based on the time it took the controller to take back the neuronal network to a stable mode. Feedback control over the non-linear model was analyzed and compared against feedback control over the linearized model. As expected feedback control over the non-linear model was more efficient on taking the neuronal network back to its stable mode by 0.76 s over 200 simulations. This was to be expected since the linearized model is an approximation. However, both modes of feedback control returned the neuronal network back to a stable behavior in less than 1.81 s. An interesting finding was that feedback control over the linearized model returned the firing rate of the most fragile node for the first time to its stable baseline faster than when applying feedback control over the non-linear model. This could be due to the fact that the transitory response from the most fragile node to the feedback control is more abrupt with a higher overpass of the stable baseline, making it harder to stabilize.

Recent efforts to design and implement closed-loop control of epileptic networks have focused on the site and stimulation parameters (Stefanescu et al., 2012; Fisher and Velasco, 2014). Ideally, electrical stimulation should be done solely on the epileptogenic network, and with accurate seizure foci localization techniques (Burns et al., 2012, 2014), it may be possible to implement micro electrode arrays at the epileptogenic brain site and measure spike trains as required by our controller scheme. However, these arrays may not be stable (i.e., stay in the same place) if chronically implanted.

Another limitation of the proposed closed-loop control design is that the stability detector input is the firing rate of the most fragile node of the network, to which feedback control is then applied. In more realistic conditions the localization of the epileptogenic source to this microscopic level is highly challenging. In this study it is assumed that the perturbation applied is known as well as the current state of all the nodes in the network. In more realistic conditions, both of these values would be unknown which represents a challenge in the design of closed-loop control for more realistic conditions.

### Conflict of interest statement

The authors declare that the research was conducted in the absence of any commercial or financial relationships that could be construed as a potential conflict of interest.
